# Hypobaric Hypoxia: Effects on Intraocular Pressure and Corneal Thickness

**DOI:** 10.1155/2014/585218

**Published:** 2014-01-16

**Authors:** Marcella Nebbioso, Stefano Fazio, Dario Di Blasio, Nicola Pescosolido

**Affiliations:** ^1^Department of Sense Organs, Sapienza University of Rome, Piazzale Aldo Moro 5, 00185 Rome, Italy; ^2^Italy Air Force Main Medical Wing, via Caluri, 1,37069 Villafranca, Verona, Italy; ^3^Department of Cardiovascular, Respiratory, Nephrology, Geriatric, and Anesthetic Sciences, Sapienza University of Rome, Piazzale Aldo Moro 5, 00185 Rome, Italy

## Abstract

*Objective*. The purpose of this study focused on understanding the mechanisms underlying ocular hydrodynamics and the changes which occur in the eyes of subjects exposed to hypobaric hypoxia (HH) to permit the achievement of more detailed knowledge in glaucomatous disease. *Methods*. Twenty male subjects, aged 32 ± 5 years, attending the Italian Air Force, were enrolled for this study. The research derived from hypobaric chamber, using helmet and mask supplied to jet pilotes connected to oxygen cylinder and equipped with a preset automatic mixer. *Results*. The baseline values of intraocular pressure (IOP), recorded at T1, showed a mean of 16 ± 2.23 mmHg, while climbing up to 18,000 feet the mean value was 13.7 ± 4.17 mmHg, recorded at T2. The last assessment was performed returning to sea level (T4) where the mean IOP value was 12.8 ± 2.57 mmHg, with a significant change (*P* < 0.05) compared to T1. Pachymetry values related to corneal thickness in conditions of hypobarism revealed a statistically significant increase (*P* < 0.05). *Conclusions*. The data collected in this research seem to confirm the increasing outflow of aqueous humor (AH) in the trabecular meshwork (TM) under conditions of HH.

## 1. Introduction

Intraocular pressure (IOP) is the consequence of the production and the outflow of aqueous humor (AH). The changes of IOP in hypobarism conditions have been over the years the subject matter of many disputed studies. In 1918, Wilmer and Berens [1] were involved in the measuring of IOP in 14 airmen, in hypobaric chamber, but they did not detect any significant IOP changes. More recently, various studies aimed at assessing changes of IOP. Some researchers detected an increase or, in contrast, a reduction of IOP, while others found no IOP variations [2–6]. The assessments of IOP in nonstandardized conditions may have had a significant influence on the data collected, representing a relevant limit. Many of these studies have been carried out as a result of climbing up to high altitude, fast acclimatizing and cold exposure, strenuous exercise, and utilizing different types of tonometers [2–7]. All the mentioned factors may have affected the measurements of IOP and the discrepancy of the results. The purpose of this study has been to better understand the mechanisms underlying ocular hydrodynamics and the changes that occur in the eyes of subjects exposed to hypobaric hypoxia (HH) to permit the achievement of more detailed knowledge and a better clinical approach in glaucomatous disease. We in fact carried out the study under standardized conditions utilizing the hypobaric chamber and the I-Care tonometer, assessing whether corneal thickness could affect the detection of IOP, as claimed by several authors [3, 8].

## 2. Materials and Methods

The research was performed according to the guidelines of the Declaration of Helsinki and Institutional Review Board approved the study protocol. Informed consent was obtained from all subjects before enrolment. Twenty male subjects, aged 32 ± 5 years, attending the Italian Air Force aerophysiological basic courses, were enrolled for this study. All individuals were free from ocular or systemic disease, including high myopia, glaucoma, macular degeneration, diabetes, multiple sclerosis, previous intraocular surgery, or trauma.

Eye examination included best-corrected visual acuity (BCVA) for far and near vision, slit lamp biomicroscopy, IOP measurements with Goldmann applanation tonometry, corneal pachymetry, gonioscopy, dilated fundus examination, and horizontal cup-disc (C/D) ratio evaluation. Minimal refraction defects were occasionally detected and BCVA for far vision was between 8/10 and 10/10 (logMAR 0.14 to −0.3). All subjects showed normal corneal thickness up to 591 *μ*m and normal electrophysiological and psychophysical tests detected with normal papillary function [9].

The research was carried out in hypobaric chamber using helmet and mask usually supplied to jet pilots connected to oxygen cylinder and equipped with a preset automatic mixer. The subjects breathed first a gas mixture at sea level (20.95% oxygen and 78.08% nitrogen). Then, simulating a condition of high altitude (18,000 to 25,000 feet), they breathed an oxygen reduced mixture (10% oxygen) while experiencing hypobarism.

The rebound portable tonometer I-Care uses a small piston with a disposable tip like a pinhead blunt, with a diameter of 0.9 mm, contact surface of 0.65 mm^2^, and a weight of 24 mg. This tip is pushed in about two milliseconds toward the corneal surface apex. IOP is directly related to the time elapsed as the tip recovers back after bouncing onto the cornea. The tonometer is nonreflexogenic and it is used without any previous contact anesthesia. It is moreover not influenced by the corneal curvature and temperature, but it is influenced by the corneal thickness. We performed measurements of the corneal thickness of the subjects joining the study by a Takagi corneal ultrasonic pachymeter. Measurements were performed first in normobaric conditions and subsequently in hypobarism.

IOP measurements were performed on twenty subjects, in both eyes, repeating the detection for each subject at least six times. IOP was assessed initially at sea level, with *P* = 760 mmHg.

After about 5 minutes, new IOP measurements were performed in both eyes at 18,000 feet above sea level (5,486 m-*P* = 380 mmHg), ascending at a rate of 4,000 feet/per minute (1,219 m/minute). After reaching an altitude of 25,000 feet, IOP measurements have been performed at 18,000 feet, during the descent (rate of 4,000 feet/per minute) and soon after returning to sea level (*P* = 760 mmHg). The subjects in the study underwent the ultrasound pachymetry in normo- and hypobarism, at 25,000 feet.

*Statistical Evaluation.* The data were analyzed by the Student's *t*-test, taking into account the average pressure values at the various stages of analysis and considering significant only those with *P* < 0.05.

## 3. Results

**Figure 1:**
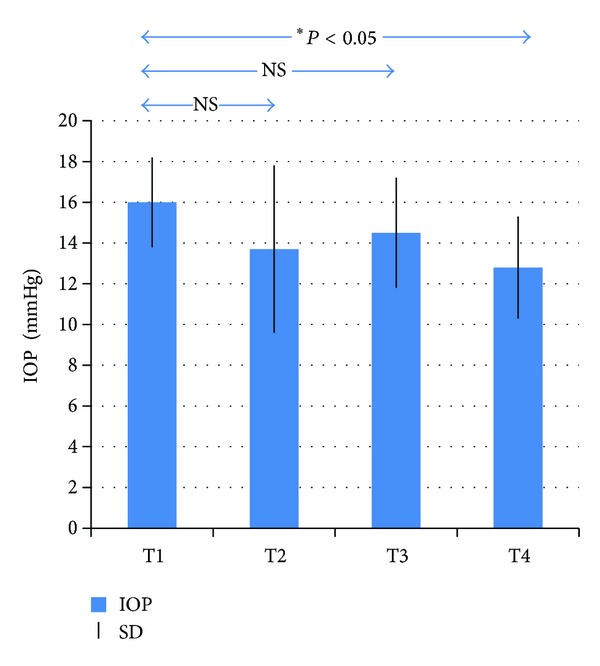
Values of tonometry measurements recorded at T1 (baseline phase to sea level), T2 (18,000 feet, phase of ascent), T3 (18,000 feet, phase of descent), and T4 (return phase to sea level). IOP: intraocular pressure (in mmHg); *statistically significant (*P* < 0.05); NS: not statistically significant; SD: standard deviation.

As highlighted in Figure 1 and Table 1, the baseline values of tonometry measurements, recorded at (T1), showed a mean IOP of 16 ± 2.23 mmHg while climbing up to 18,000 feet IOP mean value was 13.7 ± 4.17 mmHg (T2). Comparing T2 value with T1, the resulting difference was not statistically significant. IOP value of 14.5 ± 2.74 mmHg was observed in the reading of the I-Care at 18,000 feet, during the phase of descent (T3) with no statistical significance with regard to T1. The last assessment was performed returning to sea level (T4) where the mean IOP value was 12.8 ± 2.57 mmHg, with a significant change (*P* < 0.05) compared to T1 (Figure 1 and Table 1).

**Figure 2:**
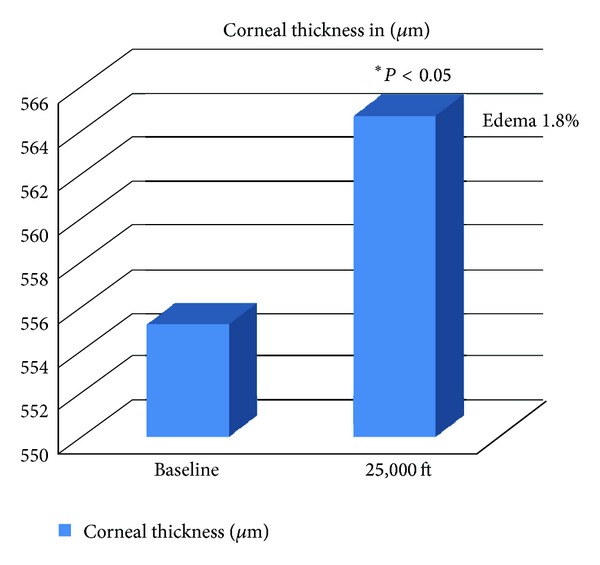
Pachymetry values related to corneal thickness in conditions of hypobarism revealed a statistically significant increase (*P* < 0.05). The average normobaric value was 555.14 ± 14.7 (SD) *μ*m versus 564.64 ± 16.5 (SD) *μ*m in hypobaric conditions, at 25,000 feet.

Pachymetry values related to corneal thickness in conditions of hypobarism revealed a statistically significant increase (*P* < 0.05). The average normobaric value was 555.14 ± 14.7 (SD) *μ*m versus 564.64 ± 16.5 (SD) *μ*m in hypobaric conditions, at 25,000 feet (Figure 2).

## 4. Discussion

The importance of IOP measurements in various environmental conditions has been over the years the subject of research, even though discordant values have been reported in the medical literature [2, 5–7, 10, 11]. Recent studies about IOP in hypobarism found out no significant changes of IOP [4], sometimes decreasing values [5, 6], and even increasing ones [2, 3].

The contradictory data among various authors are likely to be referred to the different experimental circumstances. In fact, measurements of IOP in nonstandardized conditions may have a significant influence on the resulting data, standing for a limitation. Somner et al. [2] and Bosch et al. [7] performed the tonometry while climbing up to high altitudes. Under these conditions, many factors, such as cold and strenuous exercise, seem to influence IOP values. It is well known that chilly environment [12], as well as physical exercise, [13] reduces IOP. An additional essential factor is the acclimatization process [5, 6]. This also, to a large extent, can affect the results. All the mentioned factors are not present in our study since the measurements were performed in hypobaric chamber with no other limitation.

Another factor to explain the data discrepancy is the kind of tonometer utilized in detecting IOP. The scleral rigidity may actually affect IOP assessment. This limit has been avoided in our study by means of the use of I-Care tonometer that is not affected by this factor [14]. The results previously collected have quite a few pathophysiological factors of considerable medical interest.

IOP variations between the baseline phase and the phase of return to sea level, after the step of HH was carried out, predict that an increase of a pressure-dependent AH outflow through the trabecular meshwork (TM) occurred, although it was statistically not significant during the ascent, but significant in the returning phase to normobaric conditions.

It is scarcely conceivable that these results are produced by the corneo-scleral rigidity, since IOP was measured using the I-Care tonometer that is not affected by this factor.

The corneal edema generally produces an under-evaluation of IOP, which could explain the decrease of tonometric values, as demonstrated by Simon et al. [16]. The edema causes an increase of CCT [15, 17] proportional to the water content, with loss of regular lamellar architecture [18, 19].

If this increase is now acknowledged, edema occurring during HH has only reached 1.8%, which is lower than the same physiological level of 4% at morning, when one wakes up, and it does not justify the demonstrated IOP change. Karadag et al. [15] also seem to confirm this hypothesis. According to their data, corneal thickness does not increase significantly to give reason of IOP changes. Furthermore, Somner et al. [2] stated that the changes of IOP and CCT could be analytically unrelated to each other, since corneal edema would not affect IOP and, therefore, the values obtained would be attributed to an increase of the outflow of AH.

What mechanisms could then justify IOP changes? The same mechanism discussed might validate, at least theoretically, what happens during the execution of the pneumatic trabeculoplasty. It is a technique used to reduce IOP in patients suffering from glaucoma or intraocular hypertension [20].

This procedure used to reduce IOP of those patients somehow acts the same way as hypobarism on the eye and it could be utilized to assess the outflow of the AH. The actual mechanism of the increased trabecular outflow could be represented, therefore, by an active process. Recent studies indicate that the main factors involved in changing the outflow through TM are the actomyosinic contractions of trabecular cells and the modifications of the extracellular matrix (ECM) [21–23].

In 1977 Bucci et al. [24] had already demonstrated the existence of the contractile proteins with myosin-like activities in TM cells. The contraction of TM cells is thought to adjust the flow of AH through the restructuring of TM and the variation of interaction between cells and the ECM. Any agent that increases trabecular cells contraction reduces the outflow of AH and vice versa. Actomyosin contraction seems to be depending on the phosphorylation of the myosin light chains (MLC). MLC are phosphorylated by the myosin light chain kynase (MLCK), a kinase Ca^2+^ calmodulin-dependent [25], and dephosphorylated by the myosin light chain phosphatase (MLCP) [26, 27].

MLCP activity is regulated by the action of integrin-linked kinase (ILK), protein kinase C (PKC), ZYP kinase, and especially Rho associated coil containing-protein kinase, Rho-K (ROCK) [28, 29]. ROCK determines the phosphorylation of MLCP and thus the prolongation of the actomyosinic contractile mechanism of the trabecular cells by MLCK.

The inhibition of ROCK would produce, on the other hand, the reduction of phosphorylation of MLC (MLCP remains in an active form, that is, dephosphorylated), the reduction of cellular contraction, the loss of cell-ECM adhesivity, and, therefore, the increase of the outflow of AH through TM and the consequent reduction in IOP. That statement is supported by an additional study [30] that assessed the effectiveness of forskolin for reducing IOP. Such a substance would inhibit, in fact, the activity of ROCK resulting in a reduction of IOP. Forskolin induces an increase of cAMP which would be able to inhibit Rho-A and ROCK by means of the activation of protein-kinase A (PKA). The reduction of Rho-A and its effectors, ROCK, would lead to the disassembly of actin, the increase of tissue permeability to the outflow of AH, and thus IOP reduction [30–33]. This is what would happen in the anterior chamber as a result of hypoxia-induced orbital hypobarism.

## 5. Conclusions

Changes of IOP and CCT in conditions of hypobarism have been over the years the subject matter of many disputed studies. Some of these, from a clinical point of view, seem to point out how IOP variations are mild and not very significant, while most studies gathered corresponding results regarding corneal edema. The data collected in our study seem to confirm this outcome. The possibility to better understand the mechanisms underlying ocular hydrodynamics and the changes that occur in the eye in these conditions might permit the achievement of more detailed knowledge of this subject and a better clinical approach to those subjects exposed to HH.

Unfortunately, so far, the pathophysiological basis by which these changes occur in IOP, under conditions of HH, are not completely understood. There are several hypotheses about that but probably behind this phenomenon seems to be the increasing outflow of AH in TM. A deeper knowledge of the issue could be useful to better assess the functional status of trabecular hydrodynamics in the eye. Even corneal edema during the HH is an event to be taken into account for the significant clinical implications that a prophylactic treatment could suggest.

All these evidences, however, take into consideration more detailed studies in order to improve the understanding of the mentioned mechanisms that we proposed to deal with.

## Figures and Tables

**Table 1:** Values of mean intraocular pressure (IOP) recorded at T1 (baseline phase to sea level), T2 (ascent phase to 18,000 feet), T3 (descent phase to 18,000 feet), and T4 (return phase to sea level).

Altitude (in feet)	IOP mean ± SD (in mmHg)	*P*
0 *↔*	16 ± 2.23	/
18,000 ↑	13.7 ± 4.17	T1/T2 NS
18,000 ↓	14.5 ± 2.74	T1/T3 NS
0 *↔*	12.8 ± 2.57	T1/T4 *P < 0.05

SD: standard deviation; NS: not statistically significant; *statistically significant (*P* < 0.05).
